# Immunobiologic and Antiinflammatory Properties of a Bark Extract from *Ampelozizyphus amazonicus* Ducke

**DOI:** 10.1155/2013/451679

**Published:** 2013-02-28

**Authors:** Ligia Maria Torres Peçanha, Patricia Dias Fernandes, Tatiana Jotha-Mattos Simen, Danilo Ribeiro de Oliveira, Priscilla Vanessa Finotelli, Marina Vieira Agostinho Pereira, Fernanda Ferreira Barboza, Thays da Silva Almeida, Stephanie Carvalhal, Anna Paola Trindade Rocha Pierucci, Gilda Guimarães Leitão, Luca Rastrelli, Anna Lisa Piccinelli, Suzana Guimarães Leitão

**Affiliations:** ^1^Departamento de Imunologia, Instituto de Microbiologia Paulo de Goes, Universidade Federal do Rio de Janeiro, CCS Bloco I, 2° andar, 21941-590 Rio de Janeiro, RJ, Brazil; ^2^Instituto de Ciências Biomédicas, Universidade Federal do Rio de Janeiro, CCS Bloco K, 21941-590 Rio de Janeiro, RJ, Brazil; ^3^Faculdade de Farmácia, Universidade Federal do Rio de Janeiro, CCS Bloco A 2° andar, Ilha do Fundão, 21941-590 Rio de Janeiro, RJ, Brazil; ^4^Universidade Federal do Rio de Janeiro, Instituto de Nutrição Josué de Castro, CCS Bloco JSS08, 21941-590 Rio de Janeiro, RJ, Brazil; ^5^Núcleo de Pesquisas de Produtos Naturais, Universidade Federal do Rio de Janeiro, CCS Bloco H, Rio de Janeiro, RJ, Brazil; ^6^Università di Salerno, Dipartimento di Farmácia, Via Ponte Don Melillo, 84084 Fisciano, Italy

## Abstract

*Ampelozizyphus amazonicus* is used in the treatment and prevention of malaria. The effect of an aqueous extract from this plant (SART) on the immune response was investigated by measuring immunoglobulin production induced by immunization with the antigen TNP-Ficoll in *Plasmodium chabaudi*-infected mice. SART treatment increased antigen-specific IgM and IgG levels in TNP-Ficoll-immunized mice. The B cell response during malarial infection was also modified by SART. There was an increase in total serum IgM and IgG and a decrease in the percentage of splenic plasma cells (CD138+ cells) in *P. chabaudi*-infected, SART-treated animals. SART (1, 3 or 10 mg/kg, p.o.) and the reference drug dexamethasone (5 mg/kg) were also tested in carrageenan-induced leukocyte migration to the subcutaneous air pouch (SAP). All SART doses significantly reduced leukocyte migration into the SAP. The protein concentration resulting from extravasation into the peritoneum was also significantly reduced. Our data indicate that SART possesses immunomodulatory properties, inducing an *in vivo* modification of the B lymphocyte response and anti-inflammatory properties, which are partly due to a reduction in cell migration and are most likely due to an inhibition of the production of inflammatory mediators. Preliminary HPLC-ESI-MS/MS analysis of SART shows a complex saponin profile with deprotonated molecule [M-H]^−^ ions in the range of *m/z* 800–1000.

## 1. Introduction

*Ampelozizyphus amazonicus* Ducke (Rhamnaceae) is an Amazonian medicinal plant popularly known as “saracura-mirá” that is found in the Amazon forest territories of Brazil, Venezuela, Colombia, Peru, and Ecuador. In Brazil, it is restricted to the states of Amazonas, Pará, and Roraima and grows mainly in the “terra firme” forests near waterfalls or “igarapés” [1]. An aqueous drink can be prepared from the bark and roots of *A. amazonicus*. This drink has a very bitter taste and forms abundant foam when shaken, similar to beer, which also gives rise to its other popular names: “cervejinha,” “cervejeira,” “cerveja-do-mato,” “cerveja-de-índio,” and “cerveja-de-preto” [2–5]. These properties can be explained by the high saponin content in the species. Indeed, Silva et al. [6] showed the presence of approximately 48% saponins in an aqueous extract from the roots of the plant. So far, only three saponins with a dammarane-type aglycone have been described in the literature [7, 8], as well as the presence of free triterpenes such as melaleucic acid, 3*β*,27*α*-dihydroxylup-20(29)-en-28*β*-oic acid, betulinic acid, betulin, lupeol, and phytosteroids [7–9].

Several ethnobotanical studies have shown that *A. amazonicus* is useful in the treatment and prevention of malaria [2, 3, 10–12]. Previous investigations of the antimalarial properties of an extract from this plant have shown that it does not have a direct action on *Plasmodium* blood stage forms, either *in vivo* or in red blood cell cultures [13]. However, this natural product could be effective in controlling infection induced by sporozoite forms [13]. Based on the findings that *A. amazonicus* does not have a direct effect upon blood stage forms of the protozoan, it might be possible to suggest that the control of the infection induced by this plant could be obtained by an overall augmentation of the immunological response. In fact, ethnopharmacological studies indicate both stimulatory and energetic properties for *A. amazonicus *[5, 12]. In an ethnopharmacological study conducted by our group in the “quilombola” communities of Oriximiná, State of Para, Brazil, the plant is used in the treatment of liver disorders, gastritis, inflammation of the prostate, and “inflammation of a woman” and as a fortifying tonic and aphrodisiac, among other uses [12, 14]. In the Rio Negro valley and Jaú National Park in the state of Amazonas, *A. amazonicus* is used against rheumatism and other types of pain and for the general treatment of inflammation [5, 11]. 

Based on the reported properties of this plant and its uses in folk medicine, our group suggests that *A. amazonicus* could act as an adaptogen by enhancing immune system function and could alleviate the inflammatory disorders caused by malaria. In the present work, we aimed to investigate the toxicity of *A. amazonicus* and its effects on the immune response, as well as its anti-inflammatory properties.

## 2. Material and Methods

### 2.1. Plant Material and Preparation of Extracts

*Ampelozizyphus amazonicus* Ducke was collected in August 2008, in the Brazilian Amazon region of Oriximiná (Para state), at the Pancada community (S 01°04′09.4′′ and W 056°02′40.9′′). Plants were collected as a part of a bioprospecting project in “quilombola” communities from Oriximiná that received authorization by the Brazilian Directing Council of Genetic Heritage (Conselho de Gestão do Patrimônio Genético), through Resolution number 213 (6.12.2007), published in the Federal Official Gazette of Brazil on December 27, 2007. Plants were identified by Mr. Jose Ramos (parataxonomist). A voucher specimen was deposited at the Instituto Nacional de Pesquisas da Amazônia INPA herbarium (Manaus, AM, Brazil) under the registration INPA 224161. 

Dried and ground bark (250 g) of *A. amazonicus* was used for the preparation of the extracts. The bark was submitted to extraction with boiling water (5% w/v) for 15 minutes and filtered. A second extraction was performed with boiling water (2.5%, w/v, 30 minutes). The extracts were mixed and infused into a spray-dryer nozzle unit of Büchi Mini Spray Dryer B-290 (Büchi Laboratorius-Technik AG, Switzerland). The conditions of the spray-drying process were as follows: nozzle diameter 0.3 mm, aspirator pressure 80%, flow rate 6 mL/min, inlet temperature 190 ± 3°C, and outlet temperature 88 ± 1.5°C. The atomized powder was collected by a cyclone and is designated as SART throughout the text. 

### 2.2. Estimation of Daily Dose for Animal Assays

The daily oral dose of *A. amazonicus* (SART) used was determined based on its traditional use. A drink was prepared according to the “quilombola” traditional method, as described by Oliveira et al. [14]. Briefly one tablespoon of ground bark (8.3980 g) was added to 200 mL of cold water and “shaken” seven times. The foam produced after each shaken was discarded. The extract was filtered, and a total solid yield of 0.21% (w/v) was obtained [15]. Considering that the prophylactic use of the drink (to prevent from malaria) is 0.630 g/day or 300 mL/day of extract at 0.21% (w/v) of total solid yield, the daily oral dose for an adult weighing 70 kg would be 9 mg/Kg. Therefore, all biological assays were standardized to a 10 mg/kg oral dose of SART as obtained by spray dryer.

### 2.3. HPLC-DAD Profile of SART

HPLC analysis of SART (aqueous atomized extract of *A. amazonicus, *2 mg/mL in acetonitrile : water, 95 : 5) was performed on a VWR-Hitachi Elite LaChrom system consisting of an L-2130 quaternary pump with online degasser, a Rheodyne injector (20 *μ*L loop), L-2455 diode array detector (DAD), equipped with a Zic-Hilic PEEK column (SeQuant-Merck, 250 × 4.6 mm i.d., particle size 5 *μ*m, 200 A), coupled with a security guard column (4 × 3.00 mm i.d. C-18 cartridge). The elution solvents used were ammonium acetate 10 mM, pH 5.8 corrected with acetic acid (solvent A) and acetonitrile (solvent B), and the flow rate was 0.5 mL/min, detection: DAD (210 nm). The elution was as follows: 0–10 min, isocratic 4% (solvent A): 96% (solvent B), 10.1–65 min, linear gradient where solvent B decreased from 96% to 50%. Betulinic acid from SIGMA (0.125 mg/mL in acetonitrile : water, 95 : 5) was used as standard.

### 2.4. HPLC-DAD-ESI-MS Preliminary Profile of SART

An HPLC system, including a Surveyor Autosampler, a Surveyor LC pump, and an LCQ Advantage ion-trap mass spectrometer (Thermo Finningan, San Jose, CA, USA), equipped with an electrospray ion source, was used for the preliminary analysis of SART. Chromatographic conditions were the same reported above. MS data were acquired in negative ionization mode, and the spectra were recorded in full scan (*m/z* 250–1500) and tandem mass (collision energy of 40% of the instrument maximum) scanning modes. Instrumental parameters were optimized using a purified saponin mixture isolated from SART by countercurrent chromatography (data not shown). Saponin mixture was dissolved (14 *μ*g·mL^−1^) in ACN : H_2_O (1 : 1, v/v) and infused in the ESI source at the flow rate of 5 *μ*g·min^−1^ by a syringe pump. 

### 2.5. Animals

Experiments were performed with male Swiss mice (20–25 g) obtained from Instituto Vital Brazil or BALB/c mice with ages ranging from 6 to 8 weeks supplied by the Central Animal Facility from Instituto de Microbiologia from Federal University of Rio de Janeiro. Animals were maintained in a room with controlled temperature (22 ± 2°C) with a 12 h light/dark cycle and free access to food and water. Animal care and research protocols were in accordance with the principles and guidelines adopted by the Brazilian College of Animal Experimentation (COBEA). They were approved by the Ethical Committee for Animal Research (Biomedical Science Institute/UFRJ) and received the numbers ICBDFBC-015 and IMIPPG-012.

### 2.6. Drugs and Extract Administration

Carrageenan and dexamethasone were purchased from Sigma (St. Louis, MO, USA). All drugs were dissolved in phosphate buffer saline (PBS) just before use. SART was dissolved in sterile water and administered by oral gavage at doses of 1, 3, and 10 mg/kg in a final volume of 0.1 mL. Dexamethasone (5 mg/kg) was used as reference drug and was administered via oral gavage as well. The negative control group was composed of mice given the vehicle (sterile water); significant effects due to the water per se were not observed throughout the study. The dose of dexamethasone was chosen based on previous experiments done by our group.

### 2.7. Determination of SART Toxicity *In Vitro*

Cells from the cell line A20 (3 × 10^4^) were plated in flat bottom 96-well tissue culture plates. Cultures were performed in RPMI 1640 medium supplemented with 10% fetal calf serum (Gibco-BRL, Grand Island, NY, USA), L-glutamine (2 mM), 2-ME (50 *μ*M), and gentamicin (50 *μ*g/mL). Reagents were obtained from SIGMA Chemical Co. (St Louis, MO, USA). Some cultures received different doses of SART. An incubation of 48 hours was performed at 37°C in CO_2_ Incubator (Forma Scientific, Marietta, OH, USA). Afterwards, cell viability was determined by the XTT-based viability assay [16]. Plates were read in a microplate reader (model 550 reader, Bio-Rad, Boston, MA, USA) to determine A_450_. 

### 2.8. Anti-TNP-Ficoll Immunoglobulin Production

TNP-Ficoll was obtained from Bioresearch Technologie, Inc. (Novato, CA, USA). TNP-Ficoll was diluted in PBS and administered by the intravenous injection of 50 *μ*g of the polysaccharide per animal. Oral treatment with SART was begun ten days before immunization, and each animal was treated daily with a 10 mg/kg dose of SART. The animals were bled through the tail vein weekly, and serum samples were prepared. Serum anti-TNP-Ficoll IgM and IgG titers were measured by ELISA as previously described [17]. Briefly, flexible polyvinyl chloride round bottom microtest plates (BD Falcon, BD Labware, Franklin Lakes, NJ, USA) were covered with a solution of TNP-Ficoll diluted in 1 M pH 8.3 Tris buffer. Serially diluted samples were added to the plates and were performed in duplicate. Secondary alkaline phosphatase-labeled anti-IgM or IgG antibodies (SIGMA Chemical Co. St Louis, MO, USA) were used to reveal antigen-specific antibody binding. Plates were read using a microplate reader to determine A_405_ (model 550 plate reader; Bio-Rad, Boston, MA, USA). The titer of each sample was the serum dilution giving an A_405_ reading midway on the linear portion of the titration curve. 

### 2.9. Malarial Infection and Immunoglobulin Measurement

*P. chabaudi* infection was established by the intraperitoneal injection of 10^6^ *P. chabaudi*-infected red blood cells. Serum samples were obtained as described above and were used for measuring total IgM and IgG circulating concentrations. This measurement was performed by a sandwich ELISA as previously described [18]. Unlabeled goat anti-IgM or anti-IgG antibodies (SIGMA Chemical Co., St Louis, MO, USA) were used as primary antibodies. Standard curves were set up using purified mouse IgM or IgG from ICN Biomedicals (Irvine, CA, USA). Secondary alkaline phosphatase-labeled antibodies were also obtained from SIGMA Chemical Co. The reaction was quantified by the addition of *p-*nitrophenolphosphate (SIGMA Chemical Co.), and absorbance was measured at 405 nm with a model 550 plate reader (BioRad, Boston, MA, USA). 

### 2.10. Determination of Antibody-Producing Cells by Flow Cytometry

A splenic cell suspension was obtained from individual animals and cells were diluted in RPMI 1640 medium supplemented with 5% FCS. Samples of 10^6^ cells were labeled with FITC-conjugated anti-B220 and PE-conjugated anti-CD138 antibodies (BD Biosciences, San Jose, CA, USA). The samples were washed, fixed with PBS/1% formaldehyde, and were analyzed by flow cytometry using a FACSCalibur, and data were analyzed using the CellQuest software (both are from BD Biosciences, San Jose, CA, USA).

### 2.11. Formalin Test

This procedure was similar to the method described by Gomes et al. [19]. Mice received an injection of 20 *μ*L of formalin (2.5% v/v) into the dorsal surface of the left hind paw. The time that the animal spent licking the injected paw was immediately recorded. The nociceptive and inflammatory responses consists of the following two phases: the first phase lasts until 5 min after the formalin injection (first-phase, neurogenic pain response), and the second phase occurs 15–30 min after the formalin injection (second-phase, inflammatory pain response). The animals were pretreated with oral doses of SART or vehicle for 60 min before the administration of formalin. 

### 2.12. Subcutaneous Air Pouch (SAP)

The method was adapted from Raymundo et al. [20]. Air pouches were produced by subcutaneous injection of 10 mL of sterile air into the intrascapular area of the backs of the mice. After 3 days, another 10 mL of air were injected to maintain the pouches. The animals received an injection of 0.5 mL of a sterile carrageenan suspension (1%) into the SAP three days after the last injection. The animals were pretreated with oral doses SART (1, 3, and 10 mg/kg) 60 min before carrageenan injection. The animals were killed 24 h after carrageenan injection and the cavity was washed with 2 mL of sterile PBS. The liquid in the SAP was collected and quantified. Total cell count was done in an automatic cell counter (pocH-100iV Diff, Sysmex). The exudates were centrifuged at 170 ×g for 10 min at 4°C, and the supernatants were collected and stored at −20°C until dosages. The protein content of each supernatant was determined using the BCA method (BCA Protein Assay Kit, Pierce).

### 2.13. Reduction of Spontaneous Activity

Spontaneous activity was evaluated as described by Barros et al. [21] and Figueiredo et al. [22]. Mice received 10 mg/kg of SART by oral administration and were immediately placed individually in an observation chamber with a floor that was divided into 50 squares (5 cm  ×  5 cm). The total number of squares that the mouse walked through during a 5 min period was counted. The effect of SART on locomotor performance was also tested on the rotarod apparatus as described previously [22, 23]. All animals were trained on the rotarod (3.7 cm in diameter, 8 rpm) twenty-four hours before the experiments until they could remain in the apparatus for 60 s without falling. On the day of the experiment, mice were treated with SART (at 10 mg/kg, p.o.) and tested on the rotarod from 0.5 to 3.5 h after SART administration. The number of falls from the apparatus was recorded with a stopwatch for up to 240 s.

### 2.14. Acute Toxicity

The parameters were determined as described by Lorke [24]. A single oral dose of SART (10 mg/kg) was administered to a group of ten mice (five males and five females). We observed various behaviour parameters including convulsion, hyperactivity, sedation, grooming, loss of righting reflex, increased or decreased respiration, and food and water intake over a period of 15 days. After this period, the animals were euthanized by cervical dislocation, their stomachs removed, an incision along the greater curvature was made, and the number of ulcers (single or multiple erosion, ulcer, or perforation) was counted. Hyperaemia was also evaluated. 

### 2.15. Statistical Analysis

For *in vivo* assays all experimental groups consisted of 6–10 mice. For the *in vitro* assays, all studies were done in triplicate and each protocol was repeated at least 4 times. The results are presented as mean ± S.D. Statistical significance between groups was performed by applying analyses of variance (ANOVA) followed by Bonferroni's test or Student's *t*-test for independent samples. *P* values less than 0.05 (*P* < 0.05) were considered significant.

## 3. Results and Discussion

### 3.1. HPLC-DAD Profile of SART

**Figure 1:**
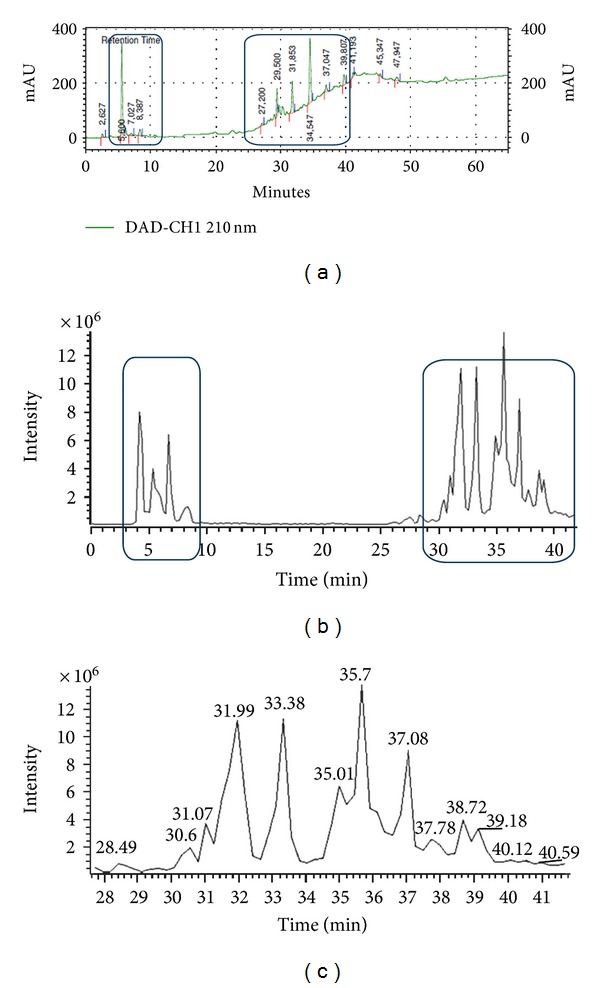
(a) HPLC-DAD chromatogram of SART (detection 210 nm) and betulinic acid at Rt = 8.387 min. (b) HPLC-DAD-ESI-MS (negative mode) chromatogram of SART. Triterpene zone between 5 and 10 min and saponin zone between 30 and 40 min (c) HPLC-DAD-ESI-MS chromatogram of SART: expansion of the saponin zone between 30 and 44 min.

In a previous study from our group [14], we demonstrated that an aqueous extract from *A. amazonicus* prepared by the traditional quilombola method contained free betulinic acid. After acid hydrolysis followed by gas chromatography-mass spectrometry analysis, the presence of a dammarane-type triterpene skeleton was also characterized in that extract [14]. In the present study, a different methodology was used for extraction, which used hot water instead of tap water, to raise the extraction yields. Because we have suggested that some of the use indications of this plant, for instance, as a tonic and for treating malaria, might be related to its properties as an adaptogen and to the immunostimulatory properties of the saponins and betulinic acid in the drink, we investigated the presence of betulinic acid in SART. The HPLC profile of SART was obtained in a Zic-Hilic column in which two different zones can be found: the first refers to triterpenic and nonpolar compounds, while the second refers to saponins and polar compounds (Figure 1(a)), thus showing the efficiency of this zwitterionic column in the separation of different compounds and polarities. The presence of betulinic acid in SART (*t*_*R*_ 8,387 min) was confirmed by standard injection.

### 3.2. HPLC-DAD-ESI-MS/MS Profile of SART

To more closely investigate the chemical composition of SART, we performed a preliminary HPLC-ESI-MS/MS analysis of the extract. The resulting (−)-MS chromatogram (Figure 1(b)) and its expansion (30–41 min) show a complex saponin profile with deprotonated molecule [M-H]^−^ ions in the range of *m/z* 800–1000. Analysis of the product ion mass spectra of [M-H]^−^ ions implied that the compounds belong to the class of triterpenoidal saponins. 

The peaks at 35.0 and 32.0 min ([M-H]^−^ at *m/z* 897 and 983) showed a fragmentation pattern that suggested the presence of triglycosides of jujubogenin. The peak at 35.0 min yielded product ions at *m/z* 765, 735, 603, and 471 corresponding to [M-H-pentose]^−^, [M-H-hexose]^−^, [M-H-pentose-hexose]^−^ and [M-H-2 × pentose-hexose]^−^. The diagnostic signals of the peaks at 32.0 min, observed in the MS^n^ spectra at *m/z* 941 ([M-H-42]^−^), 923 ([M-H-CH_3_COOH]^−^), 821 ([M-H-hexose]^−^), 633 ([M-H-42-hexose-deoxyhexose]^−^), and 471 ([M-H-42-2 × hexose-deoxyhexose]^−^), indicated the structure of an acetylated jujubogenin triglycoside. 

The peak at 36.0 min ([M-H]^−^ at *m/z* 959) showed in the MS^n^ spectra a product ion at *m/z* 817 ([M-H-C_8_H_14_O_2_]^−^) due to a McLafferty rearrangement that is characteristic of the keto-dammarane-type triterpene saponins [25]. Other product ions at *m/z* 655 ([M-H-C_8_H_14_O_2_-hexose]^−^), 509 ([M-H-C_8_H_14_O_2_-hexose-deoxyhexose]^−^), and 347 ([M-H-C_8_H_14_O_2_-2 × hexose-deoxyhexose]^−^) confirmed the presence of a trisaccharide chain. The peak at 35.7 min ([M-H]^−^ at *m/z* 973) displayed MS^n^ spectra very similar to those of the previous peak: product ions at *m/z* 817 ([M-H-C_9_H_16_O_2_]^−^), 655 ([M-H-C_9_H_16_O_2_-hexose]^−^), 509 ([M-H-C_9_H_16_O_2_-hexose-deoxyhexose]^−^) and 347 ([M-H-C_9_H_16_O_2_-2 × hexose-deoxyhexose]^−^). The difference in the *m/z* values of the [M-H]^−^ ions of these two compounds was equivalent to 14 (corresponding to a CH_2_ unit), indicating that the structural difference is in the alkyl chain of the keto-dammarane skeleton. An elucidation of the structures of these compounds is currently in progress.

### 3.3. Immunobiologic Properties of *A. amazonicus*

To evaluate the hypothesis that *A. amazonicus* could act as an adaptogen by enhancing immune system function, its immunobiological properties were examined in different *in vitro *and *in vivo* models. The daily oral dose of *A. amazonicus* used in all biological experiments was estimated based on its traditional use by the preparation of a drink according to the “quilombola” traditional method.

#### 3.3.1. Determination of SART Toxicity and Its Effect on B Cells *In Vitro*

**Figure 2:**
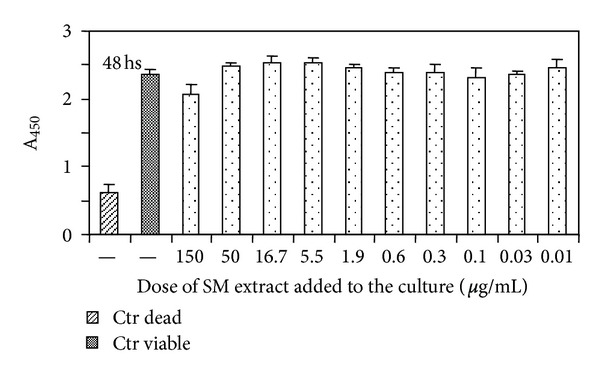
Evaluation of the toxicity of SART *in vitro* in cultured cells. The cell line A20 (3  × 10^4^ cells/well) was cultured in the presence of the indicated concentrations of SART. Control cultures were established without the addition of SART. Cultures were incubated for 48 h at 37°C in a 7% CO_2_ atmosphere. Cell viability was measured by the XTT reduction measurement. Ctr viable indicates untreated cultures incubated with medium alone. Ctr dead indicates control cultures to which 1 *μ*L of Triton X-100 was added prior to the addition of XTT. Mean values ± SD of absorbance (A_450_) are shown.

We initially measured the toxic effects of different doses of SART on A20 lymphoma cell cultures. SART did not modify cell viability in cultures incubated with this extract for either 48 h (Figure 2) or 72 h (data not shown). We also tested whether SART would modify the B lymphocyte response in culture. We employed in this investigation cultures of purified B cells stimulated with LPS and measured the stimulation of two parameters associated with B cell activation by LPS: immunoglobulin secretion and the membrane expression of the protein CD86. We did not observe any significant modification of either immunoglobulin M (IgM) secretion or CD86 cell surface expression in B cell cultures incubated with different doses of SART (data not shown). 

#### 3.3.2. Anti-TNP-Ficoll Immunoglobulin Production

**Figure 3:**
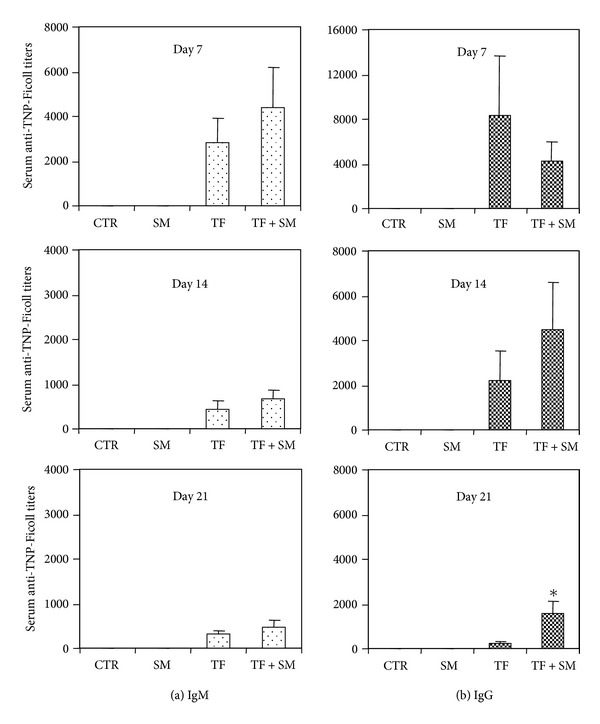
Antigen-specific immunoglobulin production in animals treated with SART. The four indicated groups were established: CTR (PBS-treated mice), SART (mice treated daily with an oral dose of SART), TF (mice immunized with TNP-Ficoll), and TF + SART (mice immunized with TNP-Ficoll and treated daily with SART). Oral treatment with SART was onset 10 days before immunization. Serum was obtained from individual mice at days 7, 14, and 21 after immunization, as indicated in the figure. Anti-TNP-Ficoll serum titers were determined by ELISA. (a) show data on IgM antibodies and (b) the one on IgG levels. Data indicate mean values ± SD of 6 animals per group. Data are representative of two independent experiments. *P ≤ 0,05 when compared to the TF group.

We next investigated the effect of SART on the *in vivo* immune response. We used immunization with the T-independent type 2 antigen TNP-Ficoll, which induces antigen-specific immunoglobulin production, as a model. This class of antigen stimulates B cells in the absence of T cell help and induces the production of both IgM and IgG [26]. Mice were immunized by intravenous injection of TNP-Ficoll, and antigen-specific immunoglobulin was measured weekly after immunization. As observed in Figure 3, oral treatment with SART induced an increase in both IgM (Figure 3(a)) and IgG (Figure 3(b)) anti-TNP-Ficoll antibody titers. 

The finding that SART treatment could amplify the response of murine B cells to a T-independent type 2 antigen suggests that components of the plant extract might have immunopotentiating effects. Saponins are the major components of the extract obtained from SART. Several studies have shown that these compounds contain adjuvant properties [27], including the ability to enhance immunoglobulin production [28] and also to stimulate the release of immune mediators and the proliferation of immune cells *in vitro *[29].

#### 3.3.3. Malaria Infection: Measurement of Serum Immunoglobulin Levels and Numbers of Splenic Antibody-Producing Cells

**Figure 4:**
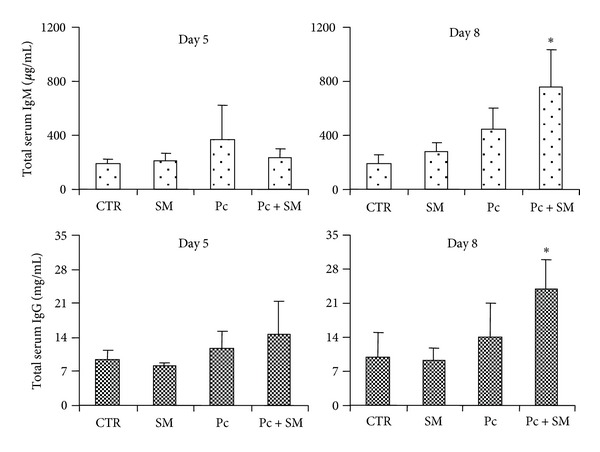
Serum total IgM and IgG levels in mice infected with *P. chabaudi* and treated with SART. The four indicated groups were set up: CTR (vehicle-treated animals), SART (mice treated with a daily oral dose of SART), Pc (animals injected with 10^6^ *P. chabaudi*-infected red blood cells), and Pc + SART (*P. chabaudi*-infected SART-treated mice). Groups of 8 animals were used and two independent experiments were performed. Serum was obtained at the indicated periods after infection and total IgM (top panels) and IgG (bottom panels) were determined by ELISA. Data indicate mean immunoglobulin levels ± SD. *P ≤ 0,05 when compared to the Pc group.

**Figure 5:**
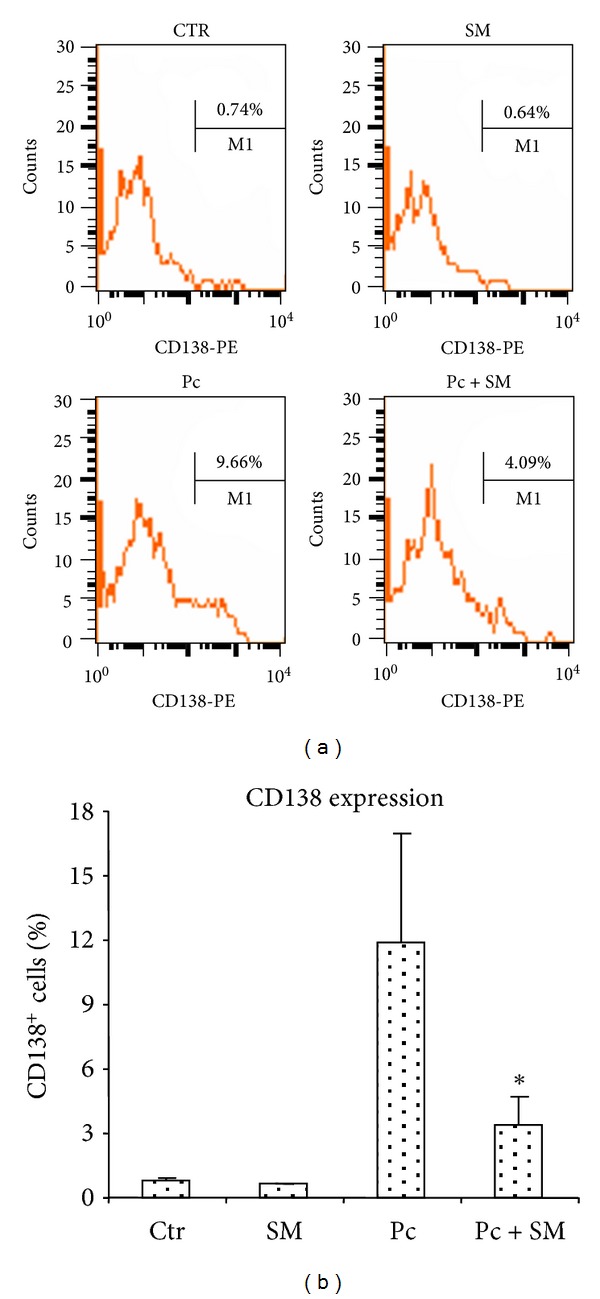
Levels of antibody-producing (CD138^+^) cells in the spleen of *P. chabaudi*-infected animals treated with SART. Four different groups: CTR, SART, Pc, and Pc + SART were established as detailed in Figure 4. Splenic cells were obtained from individual animals and 10^6^ cells were labeled with anti-B220-FITC and anti-CD138-PE antibodies. Samples were run in a FACScalibur Flow Cytometer. Data shown in (a) indicate a typical profile of CD138^+^ cells in the B220^+^ lymphoid population in each experimental group. The percentage of CD138^+^ cells is included in each graphic. Data in (b) indicate the mean value ± SD of the percentage of CD138^+^ cells from each group. Six animals were used per group and data are representative of two independent experiments. *P ≤ 0,05 when compared to the Pc group.

As described above, SART treatment is popularly used to control malaria infection. The role of B cells in this infection was previously investigated, and it was shown that mice deficient in mature B cells had reduced primary acute infections but were unable to eliminate parasites, indicating that B cells are required for final parasite clearance [30]. It was also described that *Plasmodium*-infected animals showed an increase in circulating antibody levels when compared to normal animals [31]. Therefore, we next investigated the effects of SART treatment on the course of the B cell response in *P. chabaudi*-infected mice. We examined whether SART treatment could induce a modification in immunoglobulin production during infection. Treatment with SART induced an increase in the levels of circulating total IgM and IgG in *P. chabaudi*-infected mice (Figure 4). We next investigated the levels of antibody-secreting cells in the spleen of *P. chabaudi*-infected SART-treated mice. To perform this study, we measured the percentage of cells expressing the surface protein CD138, a marker for antibody-secreting cells [32]. Despite the increase in circulating immunoglobulin levels, there was a decrease in the number of antibody-producing plasma cells in the spleens of *P. chabaudi*-infected, SART-treated mice 8 days after infection when compared to infected untreated animals (Figure 5).

Taken together, our data indicate that SART modulates malaria infection because SART increases immunoglobulin production during infection and regulates the appearance of antibody-secreting cells. The relevance of this phenomenon on resistance and the progression of infection need to be further analyzed. 

Due to the effect of SART on immunoglobulin production, the saponins may have antimalarial properties that are potentiated through other mechanisms. Dammarane-type saponins have shown potent hepatoprotective effects in different models [33–35], and these effects could justify the use of this plant for the treatment of liver disorders and malaria infection. 

### 3.4. Evaluation of the Anti-Inflammatory Activity of SART

Based on the ethnopharmacological information that *A. amazonicus* is indicated to treat “inflammation of the prostate,” “inflammation of a woman,” and liver disorders that could be related to inflammatory processes, we decided to evaluate the possible anti-inflammatory activity of this plant in different models.

#### 3.4.1. Formalin Test

The injection of formalin (2.5%) induces a biphasic licking response in the injected paw of mice. The first phase occurs until 5 min after injection, and the second phase occurs between 15 and 30 min after formalin injection. Pretreatment of mice with 1, 3, or 10 mg/kg SART did not reduce the time that the animal spent licking the formalin-injected paw in the first or second phase (data not shown). 

#### 3.4.2. Subcutaneous Air Pouch (SAP)

**Figure 6:**
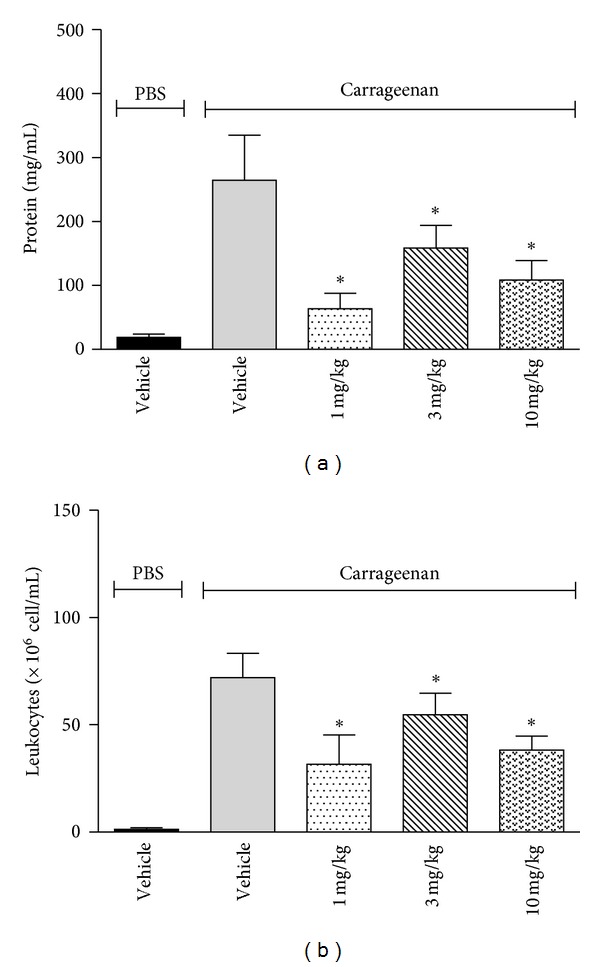
Effect of SART on leukocyte migration (a) and protein extravasation (b) into the subcutaneous air pouch. Animals were pretreated with oral administration of different doses of SART 1 h prior to carrageenan (1%) injection into the SAP. The results are presented as mean ± S.D. (*n* = 6 − 10) of number of leukocyte (×10^6^ cell/mL) (a) or protein (mg/mL) (b). Statistical significance was calculated by ANOVA followed by Bonferroni's test. *P < 0.05 when comparing latex-treated mice to the vehicle-treated group; ^#^ *P* < 0.05 when comparing vehicle-treated mice to the PBS-treated group.

To evaluate the possible anti-inflammatory activity of SART, the carrageenan-induced inflammation model in the SAP was used. This model involves synovial inflammation caused by carrageenan injection into the air pouch that forms in the back of mice. This procedure induces the proliferation of cells that stratify on the surface. The injection of carrageenan drastically increased the exudate volume into the pouch up to twice the level of mice that received PBS in the SAP. In the absence of SART, the numbers of leukocytes in the carrageenan-injected air pouch exudates were markedly increased up to 57-fold higher than control levels Figure 6(a); (1.25 ± 1.1 × 10^6^ cells/mL versus 72 ± 12.4 × 10^6^ cells/mL). These numbers were markedly reduced by approximately 56%, 24%, 77.6%, and 47% by pretreatment with increasing doses of SART. The pretreatment of mice with SART significantly reduced the volume of exudate recovered from the air pouches. Carrageenan treatment also caused a 14-fold increase in the exudate protein concentration, and the pretreatment of mice with SART significantly inhibited carrageenan-induced protein leakage with all doses tested Figure 6(b). Using an *in vivo *air pouch inflammation model, we showed that carrageenan increased both the exudate volume and the exudate protein concentration, which indicates vascular leakage of the serum contents. Carrageenan is also an important chemotactic agent because it induces the migration of inflammatory cells such as neutrophils and macrophages [36]. In this study, SART reduced leukocyte migration, exudate volume, and protein extravasation, even at a dose of 1 mg/kg, suggesting a suppression of vascular leakage. These results show an anti-inflammatory effect for SART, which was demonstrated by reduced cell migration and liquid and protein extravasation. Acute inflammation, such as carrageenan-induced cell migration, involves the synthesis or release of mediators at the injured site. These mediators include prostaglandins, particularly those of the E series, histamine, bradykinins, leukotrienes, and serotonin, all of which also cause pain and fever [37]. Inhibiting these mediators to prevent them from reaching the injured site and exerting their pharmacological effects will normally ameliorate inflammation and other symptoms. This study has shown that SART possesses the ability to significantly affect cell migration and protein leakage induced by carrageenan, suggesting a possible modulation of some events related to inflammatory process. Because the carrageenan-induced inflammation model is a significant predictive test for anti-inflammatory agents that act on mediators of acute inflammation [38, 39], these results are an indication that SART may be effective in treating acute inflammatory disorders. Moreover, this effect could explain the reduction in the inflammatory processes that accompany malaria, which have already been discussed for other plant species used in the treatment of this disease [40]. Triterpene saponins have demonstrated anti-inflammatory properties through the inhibition of iNOS and proinflammatory cytokine expression [41]. These compounds exhibited significant xanthine oxidase inhibitory activity, preventing the overaccumulation and deposition of uric acid in the joints, which leads to painful inflammation [42]. 

More assays, such as assays that determine the dosing of some inflammatory mediators, are underway to reveal the exact mechanisms of the anti-inflammatory action of SART.

### 3.5. Acute Toxicity and Reduction of Spontaneous Activity

The oral administration of a 10 mg/kg dose of SART did not induce any toxic effects. No behavioral alterations, lesions, or gastric bleeding was observed. Additionally, no signs of intoxication, including convulsion, death, or gastric ulcer, were observed, even after 5 days of a single dose (data not shown). It is noteworthy that the oral administration of SART did not show any gastric disorders because the irritation of the gastric mucosa is an important side effect that is expected with the oral use of saponin-containing herbs such as Horse Chestnut, *Ruscus* [43]. 

## 4. Conclusions

HPLC-ESI-MS/MS analysis of SART showed a complex dammarane-type saponin profile with molecular weights in the range of *m/z* 800–1000. The biological results shown here indicate that SART possesses immunomodulatory properties that induce an *in vivo* modification of the B lymphocyte response, as well as anti-inflammatory properties, partly due to a reduction in cell migration and most likely due to an inhibition of inflammatory mediator production. Therefore, the results presented in this work support our hypothesis that *A. amazonicus* could act as an adaptogen by enhancing immune system function and could mitigate the inflammatory disorders caused by malaria. Future studies on the chemical structures of the saponins isolated from SART and their roles in the B cell response, resistance to malaria infection, and the anti-inflammatory activity of the extract will be important.
